# Female adipose tissue-specific *Bscl2* knockout mice develop only moderate metabolic dysfunction when housed at thermoneutrality and fed a high-fat diet

**DOI:** 10.1038/s41598-018-36078-9

**Published:** 2018-12-14

**Authors:** George D. Mcilroy, Sharon E. Mitchell, Weiping Han, Mirela Delibegović, Justin J. Rochford

**Affiliations:** 1 The Rowett Institute, University of Aberdeen, Aberdeen, UK; 2 Institute of Biological and Environmental Sciences, University of Aberdeen, Aberdeen, UK; 3 Laboratory of Metabolic Medicine, Singapore Bioimaging Consortium, Agency for Science, Technology and Research (A*STAR), Singapore, Singapore; 4 Institute of Medical Sciences, University of Aberdeen, Aberdeen, UK

## Abstract

Mutations affecting the *BSCL2* gene cause the most severe form of congenital generalised lipodystrophy. Affected individuals almost completely lack adipose tissue and suffer from severe diabetes and metabolic complications. Likewise, mice lacking *Bscl2* in all tissues have dramatically reduced adipose mass, glucose intolerance and hyperinsulinaemia. However, male adipose tissue-specific *Bscl2* knockout mice fail to develop the metabolic dysfunction observed in *Bscl2* null mice and *BSCL2* deficient patients, despite a similar generalised lack of adipose tissues. Clinical reports indicate gender differences frequently exist in cases of lipodystrophy, with female patients more adversely affected than male patients. We therefore generated and characterised female mice lacking *Bscl2* specifically in adipose tissue (Ad-B2^(−/−)^). We show that female Ad-B2^(−/−)^ mice also develop early-onset lipodystrophy when fed a chow diet and are maintained under standard housing conditions (21 °C) or thermoneutrality (30 °C). Despite this, female Ad-B2^(−/−)^ mice fail to develop severe metabolic dysfunction. Only when female Ad-B2^(−/−)^ mice are maintained at thermoneutrality and fed a high-fat diet do subtle alterations to metabolic homeostasis manifest. This is despite a striking inability to expand adipose mass. Our findings provide further evidence that loss of *Bscl2* in non-adipose tissues may contribute to the severity of metabolic dysfunction in this condition.

## Introduction

Lipodystrophy is a term that is used to describe conditions featuring altered adipose tissue mass or distribution^1^. Under normal circumstances, adipose tissue depots are diverse and display a number of distinct functions, depending on their anatomical location^2^. However, the primary function of adipose tissue is to act as a storage site, safely sequestering excessive energy intake in the form of triglyceride and releasing this in times of metabolic need. In conditions of lipodystrophy, this storage capacity is severely impaired as a result of abnormal adipose tissue development and/or maintenance^1^. This leads to the inappropriate accumulation of lipids into non-adipose tissues, which are not specifically adapted for this purpose. Consequently, patients suffering from either genetic or acquired forms of lipodystrophy manifest abnormalities in metabolic homeostasis, such as hepatic steatosis, hypertriglyceridemia and insulin resistance^3^. The severity of metabolic dysfunction that presents in patients with lipodystrophy can vary due to the extent and site of adipose tissue loss as well as gender^1^.

Congenital generalized lipodystrophy (CGL) is characterised by the near complete lack of adipose tissue, which leads to the development of severe metabolic dysfunction^4^. CGL is a rare genetic disorder that is inherited in an autosomal recessive manner. Four subtypes of CGL have been identified, which result from mutations in *AGPAT2* (CGL1)^5^, *BSCL2* (CGL2)^6^, *CAV1* (CGL3)^7^ and *PTRF* (CGL4)^8^. Of the four types, the most severe form of CGL observed in humans is caused by mutations that affect the *BSCL2* gene (CGL2). *BSCL2* encodes a trans-membrane protein called seipin that is located in the endoplasmic reticulum^9,10^. *In vitro* studies have revealed that seipin is critical for the development of adipocytes, as knockdown of *Bscl2* in pre-adipocyte cell lines leads to the inhibition of adipogenesis^11,12^. Seipin has also been shown to be capable of forming homo-oligomeric structures^13,14^. These appear to act as molecular scaffolds, capable of binding important regulators of triacylglycerol and glycerophospholipid synthesis such as LIPIN1, AGPAT2 and GPAT3^15–17^.

The generation of *in vivo* models to investigate CGL2 has also provided further insights and understanding of this severe genetic disorder. To date, four independent groups have generated global *Bscl2* knockout mouse models^18–21^. All four animal models are lipodystrophic and develop severe hepatic steatosis, glucose intolerance and insulin resistance. Therefore *Bscl2* null mice almost entirely recapitulate the metabolic disturbances observed in the human condition^22^. Tissue-specific knockout models have also been generated to try and further understand and characterise this condition^23–25^. We recently reported that adipose tissue-specific ablation of *Bscl2* from birth is sufficient to cause early-onset generalised lipodystrophy in male mice^21^. Surprisingly however, this model failed to develop the severe metabolic dysfunction that is observed in global *Bscl2* knockout mice, even when challenged with a high-fat diet. Interestingly, it is reported that women are typically more severely affected by lipodystrophy^1^. Indeed, clinical evidence suggests gender differences in genetic forms of lipodystrophy such as CGL2 or familial partial lipodystrophy (Dunnigan type)^26,27^, where female patients are more affected by metabolic complications than male patients. For this reason, we investigated whether female mice that lack seipin specifically in adipose tissue would develop metabolic dysfunction, despite this not being apparent in our previous study of male mice^21^. In addition, significant preservation of brown adipose mass has been observed in all *Bscl2* null mouse models, as well as in our adipose specific *Bscl2* knockout mice^18–21^. Therefore, we also examined whether the thermogenic activity of this tissue might protect mice from the metabolic consequences of lipodystrophy due to adipose specific *Bscl2* ablation.

In this study, we have generated and characterised female mice that lack *Bscl2* specifically in developing and mature adipocytes using the *Adipoq*-Cre model. Our data reveal that loss of seipin specifically in adipose tissue of female mice is sufficient to cause generalised lipodystrophy early in life. However, severe metabolic dysfunction is not apparent in chow fed mice when maintained under standard housing temperatures (21 °C) or when placed at thermoneutral housing conditions (30 °C). Signs of metabolic dysfunction only begin to manifest in aged female mice lacking *Bscl2* specifically in adipose tissue, after being challenged with a high-fat diet when housed under thermoneutral conditions. This is despite an inability to expand adipose mass which is apparent in control mice under these conditions. These findings provide further evidence that ablation of *Bscl2* in adipose tissue alone appears to be insufficient to recapitulate the full metabolic phenotype observed in global *Bscl2* knockout mice and patients that suffer from CGL2.

## Results

### Adipose tissue-specific deletion of *Bscl2* is sufficient to cause lipodystrophy in female mice

**Figure 1:**
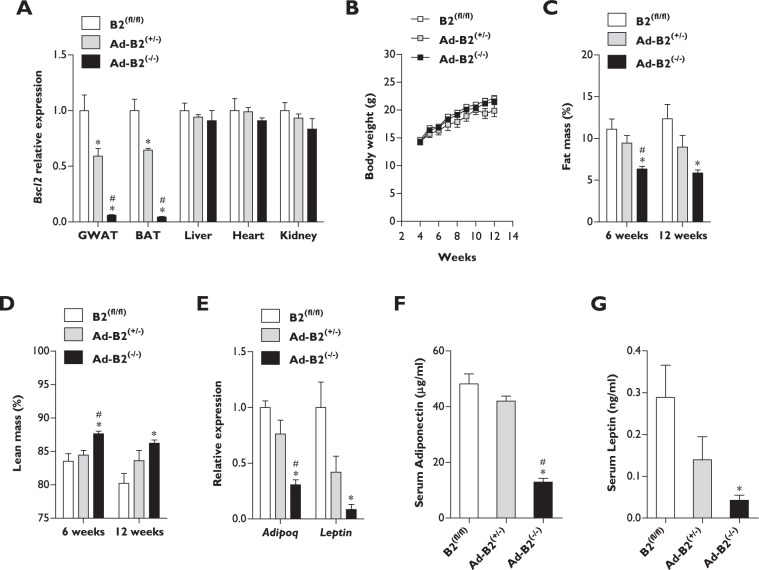
Adipose specific ablation of *Bscl2* in female mice is sufficient to cause lipodystrophy. (**A**) *Bscl2* mRNA levels of sixteen week old B2^(fl/fl)^, Ad-B2^(+/−)^ and Ad-B2^(−/−)^ mice at standard housing temperatures (21 °C) in GWAT, BAT, liver, heart and kidney (n = 3–4). (**B**) Body weight progression of B2^(fl/fl)^, Ad-B2^(+/−)^ and Ad-B2^(−/−)^ mice (n = 4–8). Fat mass (**C**) and lean mass (**D**) levels assessed by Echo-MRI and normalised to body weight in B2^(fl/fl)^, Ad-B2^(+/−)^ and Ad-B2^(−/−)^ mice at six and twelve weeks of age (n = 4–8). Relative GWAT mRNA levels (**E**) and circulating serum levels of adiponectin (**F**) and leptin (**G**) in sixteen week old B2^(fl/fl)^, Ad-B2^(+/−)^ and Ad-B2^(−/−)^ mice fasted for five hours (n = 4). All data are presented as the mean ± SEM, *p < 0.05 vs B2^(fl/fl)^, ^#^p < 0.05 vs Ad-B2^(+/−)^.

Female mice lacking *Bscl2* specifically in developing and mature adipocytes (Ad-B2^(−/−)^ mice) were generated by crossing *Bscl2* flox’d mice (B2^(fl/fl)^) with heterozygous *Bscl2* flox’d mice (B2^(fl/+)^) also carrying Cre recombinase driven by the *Adipoq* promoter, as previously described^21^. The specificity of *Bscl2* ablation was confirmed by qPCR. *Bscl2* mRNA transcript levels were significantly decreased by ~40% in Ad-B2^(+/−)^ and ~95% in Ad-B2^(−/−)^ female mice compared to B2^(fl/fl)^ female mice in both gonadal white adipose tissue (GWAT) and brown adipose tissue (BAT). However, no significant differences in *Bscl2* gene expression levels were detected in the liver, heart or kidney between any of the genotypes (Fig. 1A). Similarly to findings observed in male Ad-B2^(−/−)^ mice^21^, no significant differences in body weight were detected between any genotype when female mice were fed a standard rodent chow diet and maintained under standard housing temperature (21 °C) conditions (Fig. 1B). Echo-MRI analysis revealed that female Ad-B2^(−/−)^ mice had significantly decreased fat mass levels (Fig. 1C) and significantly increased lean mass levels (Fig. 1D) as a percentage of bodyweight compared to both B2^(fl/fl)^ and Ad-B2^(+/−)^ mice at six weeks of age. Significant changes in fat mass and lean mass as a percentage of body weight were also observed at twelve weeks of age, but this was only apparent between B2^(fl/fl)^ and Ad-B2^(−/−)^ female mice (Fig. 1C,D). Absolute lean mass was not found to be significantly different at six weeks of age, however female Ad-B2^(−/−)^ mice had significantly increased lean mass compared to Ad-B2^(+/−)^ mice at twelve weeks of age (Fig. S1A). Gene expression analysis of GWAT in sixteen week old female mice revealed significant decreases in *Adipoq* in Ad-B2^(−/−)^ mice compared to B2^(fl/fl)^ and Ad-B2^(+/−)^ mice and decreases in *Leptin* were found in Ad-B2^(−/−)^ mice compared to B2^(fl/fl)^ controls (Fig. 1E). Circulating serum levels of adiponectin and leptin in mice fasted for five hours showed similar alterations to those seen at the mRNA transcript level (Fig. 1F,G). The findings presented therefore indicate that similarly to that observed in male Ad-B2^(−/−)^ mice^21^, ablation of *Bscl2* specifically in developing and mature adipocytes in female mice is sufficient to cause lipodystrophy.

### Female Ad-B2^(−/−)^ mice do not develop severe metabolic dysfunction

**Figure 2:**
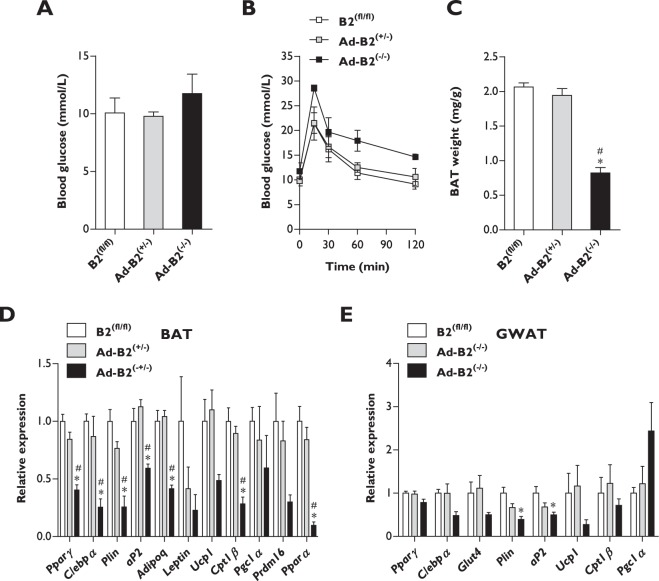
Metabolic consequences of adipose tissue-specific *Bscl2* deletion in female mice. Five hour fasted blood glucose levels (**A**) and glucose tolerance test (**B**) in B2^(fl/fl)^, Ad-B2^(+/−)^ and Ad-B2^(−/−)^ mice at ten weeks of age (n = 4). (**C**) BAT weight normalised to body weight in sixteen week old mice (n = 3–4). mRNA levels of white and brown markers in (**D**) BAT (n = 3–4) and (**E**) GWAT (n = 4) in sixteen week old mice. All data are presented as the mean ± SEM, *p < 0.05 vs B2^(fl/fl)^, ^#^p < 0.05 vs Ad-B2^(+/−)^.

Global *Bscl2* knockout mouse models are lipodystrophic and develop severe metabolic dysfunction, similarly to patients with pathogenic mutations in *BSCL2*^18–21^. We recently reported that adipose tissue-specific ablation of *Bscl2* in male mice is sufficient to cause lipodystrophy, yet severe metabolic dysfunction does not manifest in this model^21^. To determine if this was also the case in Ad-B2^(−/−)^ female mice, we performed glucose tolerance tests at ten weeks of age in chow fed mice maintained under standard housing temperatures (21 °C). In mice fasted for five hours, basal blood glucose levels were not found to be significantly different between genotypes (Fig. 2A). Although Ad-B2^(−/−)^ female mice appeared to exhibit a slight impairment in the ability to clear a glucose bolus during the glucose tolerance test compared to B2^(fl/fl)^ and Ad-B2^(+/−)^ controls (Fig. 2B), this failed to reach statistical significance at any time point. When serum parameters were examined at sixteen weeks of age in mice fasted for a period of five hours, no significant differences in circulating insulin, glucose or triglyceride levels were observed (Fig. S1B–D). Quantitative insulin sensitivity check index (QUICKI) analysis also indicated that female Ad-B2^(−/−)^ mice were not insulin resistant compared to control animals (Fig. S1E). Additionally, no significant differences were detectable in liver triglyceride levels between any of the genotypes (Fig. S1F). When interscapular BAT mass was examined however, female Ad-B2^(−/−)^ mice had significantly decreased amounts of this adipose tissue depot (~60%) compared to B2^(fl/fl)^ and Ad-B2^(+/−)^ mice (Fig. 2C). The extent of BAT loss in this model was more severe than that previously observed in male Ad-B2^(−/−)^ mice where BAT mass was reduced by only ~19% compared to Ad-B2^(+/−)^ mice^21^. We next examined if ablation of *Bscl2* in BAT caused any alterations to gene expression levels of white and brown adipose tissue markers. Significant decreases in *Pparγ*, *C/ebpα*, *Plin*, *aP2*, *Adipoq*, *Cpt1β* and *Pparα* were observed in BAT from Ad-B2^(−/−)^ mice compared to both B2^(fl/fl)^ and Ad-B2^(+/−)^ female mice (Fig. 2D). Transcript levels of *Leptin*, *Ucp1*, *Pgc1α*, and *Prdm16* also showed a similar trend to be decreased in Ad-B2^(−/−)^ mice, but were not found to be significantly different. Both global *Bscl2* knockout mice and male Ad-B2^(−/−)^ mice have been shown to display elevated gene expression levels of thermogenic markers in residual epididymal white adipose tissue (EWAT) depots^19,21^. To determine if this was also true in female mice, markers of white and brown adipose tissue were examined in the GWAT depot. Levels of *Plin* and *aP2* were significantly decreased in Ad-B2^(−/−)^ mice but only compared to B2^(fl/fl)^ controls, however no significant changes were found between any genotype for *Pparγ*, *C/ebpα* or *Glut4* (Fig. 2E). Unlike the increased expression of markers of thermogenesis such a *Ucp1*, *Cpt1β* or *Pgc1α* in EWAT observed in male Ad-B2^(−/−)^ mice^21^, we saw no significant increases in the expression of these genes in the GWAT of Ad-B2^(−/−)^ female mice (Fig. 2E). The data reveal that, similarly to male Ad-B2^(−/−)^ mice, adipose tissue specific ablation of *Bscl2* in female mice is insufficient to cause severe metabolic dysfunction. However, gender differences are apparent as BAT appears to be more affected by *Bscl2* loss than in male mice, and female mice do not display increases in markers of thermogenesis in residual GWAT depots.

### Housing female Ad-B2^(−/−)^ mice at thermoneutrality does not impair glucose tolerance

**Figure 3:**
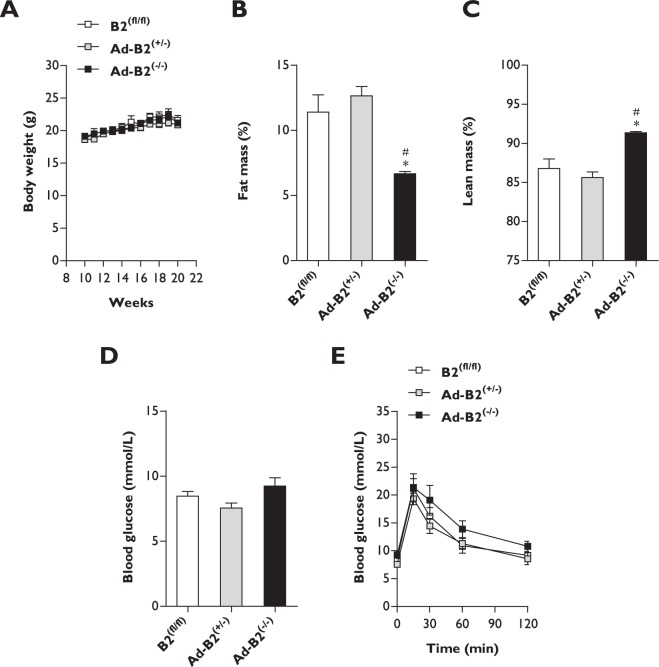
Female Ad-B2^(−/−)^ mice housed at thermoneutrality are not glucose intolerant. (**A**) Body weight progression of B2^(fl/fl)^, Ad-B2^(+/−)^ and Ad-B2^(−/−)^ mice place at thermoneutrality (30 °C) at eleven weeks of age (n = 5–6). Fat mass (**B**) and lean mass (**C**) levels assessed by DEXA normalised to body weight in B2^(fl/fl)^, Ad-B2^(+/−)^ and Ad-B2^(−/−)^ mice after eight weeks at thermoneutrality (n = 5–6). Five hour fasted blood glucose levels (**D**) and glucose tolerance test (**E**) in B2^(fl/fl)^, Ad-B2^(+/−)^ and Ad-B2^(−/−)^ mice after nine weeks at thermoneutrality (n = 5–6). All data are presented as the mean ± SEM, *p < 0.05 vs B2^(fl/fl)^, ^#^p < 0.05 vs Ad-B2^(+/−)^.

Mice that are maintained at standard housing temperatures (21 °C) are under a constant thermal stress. They are able to maintain their core body temperature by increasing rates of energy metabolism through adaptive thermogenesis^28^. In order to determine if the residual BAT in Ad-B2^(−/−)^ female mice was offering any protection from developing glucose intolerance, we placed a separate cohort of eleven week old B2^(fl/fl)^, Ad-B2^(+/−)^ and Ad-B2^(−/−)^ female mice at thermoneutral housing conditions (30 °C) for a period of nine weeks. Once again, we found no significant differences in body weight between any of the genotypes, both prior to and throughout the nine week housing period at thermoneutrality (Fig. 3A). After eight weeks of housing at thermoneutrality, dual-energy X-ray absorptiometry (DEXA) analysis was performed. The findings revealed that Ad-B2^(−/−)^ female mice had significantly decreased fat mass levels (Fig. 3B) and significantly increased lean mass levels (Fig. 3C) as a percentage of body weight compared to both B2^(fl/fl)^ and Ad-B2^(+/−)^ controls. Absolute lean mass was also significantly increased in Ad-B2^(−/−)^ female mice compared to Ad-B2^(+/−)^ mice (Fig. S1G). Thus, Ad-B2^(−/−)^ female mice are similarly lipodystrophic when housed under thermoneutral temperature conditions. After nine weeks at thermoneutrality, five hour fasted basal blood glucose levels were again found to not be significantly different (Fig. 3D) and glucose tolerance tests once again indicated that Ad-B2^(−/−)^ female mice were not glucose intolerant compared controls (Fig. 3E). Overall, the data presented reveal that lipodystrophic female Ad-B2^(−/−)^ mice fed a standard rodent chow diet that are housed under thermoneutral conditions fail to develop glucose intolerance. This is in stark contrast to global *Bscl2* knockout mouse models that develop severe metabolic dysfunction when fed a standard chow diet and are kept under standard housing temperatures.

### High-fat diet fed Ad-B2^(−/−)^ female mice housed at thermoneutrality resist diet induced weight gain

**Figure 4:**
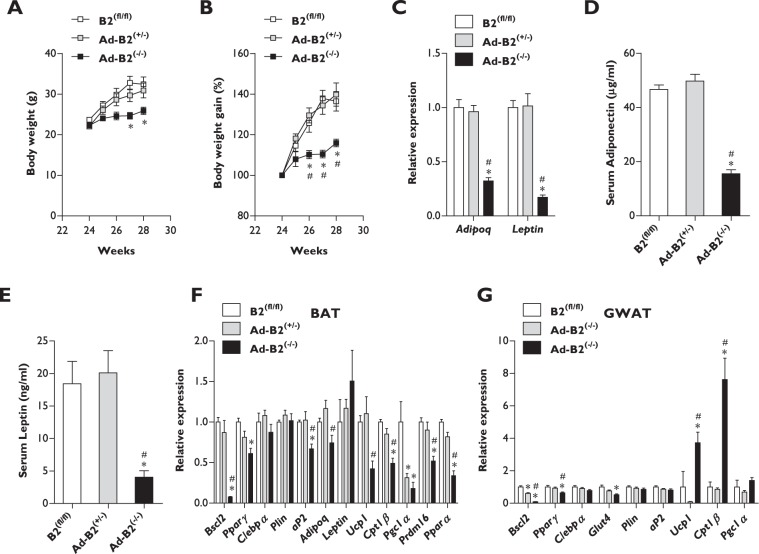
High-fat diet fed female Ad-B2^(−/−)^ mice resist weight gain at thermoneutrality. Body weight progression (**A**) and percentage of body weight gain (**B**) in twenty-four week old B2^(fl/fl)^, Ad-B2^(+/−)^ and Ad-B2^(−/−)^ female mice housed at thermoneutrality and fed a high-fat diet for four weeks (n = 5–6). Relative GWAT mRNA levels (**C**) and circulating serum levels of adiponectin (**D**) and leptin (**E**) in twenty-eight week old B2^(fl/fl)^, Ad-B2^(+/−)^ and Ad-B2^(−/−)^ mice fasted for 5 hours (n = 5–6). mRNA levels of white and brown markers in (**F**) BAT (n = 4–6) and (**G**) GWAT (n = 5–6) in twenty-eight week old B2^(fl/fl)^, Ad-B2^(+/−)^ and Ad-B2^(−/−)^ mice. All data are presented as the mean ± SEM, *p < 0.05 vs B2^(fl/fl)^, ^#^p < 0.05 vs Ad-B2^(+/−)^.

As glucose tolerance did not worsen by housing female Ad-B2^(−/−)^ mice at thermoneutrality, we next challenged them by feeding them a high-fat diet (HFD, 60% kcal from fat) for four weeks. Similarly to male Ad-B2^(−/−)^ mice fed a HFD at standard housing temperatures^21^, Ad-B2^(−/−)^ female mice resisted diet induced weight gain compared to B2^(fl/fl)^ and Ad-B2^(+/−)^ controls (Fig. 4A). This was even more apparent when represented as a percentage of body weight gain, as Ad-B2^(−/−)^ mice were significantly different compared to both B2^(fl/fl)^ and Ad-B2^(+/−)^ mice at two, three and four weeks of HFD feeding (Fig. 4B). Once again, gene expression analysis of GWAT in twenty-eight week old female mice revealed significant decreases in *Adipoq* and *Leptin* in Ad-B2^(−/−)^ mice compared to both B2^(fl/fl)^ and Ad-B2^(+/−)^ female mice (Fig. 4C). Additionally, circulating serum levels of adiponectin and leptin in mice fasted for five hours showed similar significant alterations to those seen at the mRNA transcript level (Fig. 4D,E). This indicates that Ad-B2^(−/−)^ mice continue to display severe disruption to adipose tissue development even when fed a HFD at thermoneutral conditions. When interscapular BAT mass was examined, the weight of this adipose depot had increased from ~2 mg/g, seen at standard housing temperatures, to ~3 mg/g at thermoneutrality in both B2^(fl/fl)^ and Ad-B2^(+/−)^ female mice. This is consistent with enhanced lipid accumulation in this anatomical BAT depot and accumulation of WAT-like cells. BAT mass in female Ad-B2^(−/−)^ mice had also increased to levels that were now not significantly different to control animals (Fig. S1H). Examination of white adipose tissue markers in BAT revealed that, *C/ebpα*, *Plin*, *aP2*, *Adipoq* and *Leptin* were not as dramatically impaired in Ad-B2^(−/−)^ mice when compared with the differences observed under standard housing temperatures. This was despite the almost complete ablation of *Bscl2* (Fig. 4F). Brown adipose tissue markers such as *Ucp1*, *Cpt1β, Pgc1α*, *Prdm16* and *Pparα* however were similarly impaired or even more affected (Fig. 4F) than previously observed under standard housing temperatures. Gene expression analysis in GWAT revealed only small decreases to *Pparγ* and *Glut4* in Ad-B2^(−/−)^ mice but no significant differences were observed in the expression of *C/ebpα*, *Plin*, *aP2* or *Pgc1α*. Interestingly, however, significant increases in *Ucp1* and *Cpt1β* were now seen in Ad-B2^(−/−)^ female mice compared to both B2^(fl/fl)^ and Ad-B2^(+/−)^ control mice (Fig. 4G).

### High-fat diet fed Ad-B2^(−/−)^ female mice housed at thermoneutrality only partially develop metabolic dysfunction

**Figure 5:**
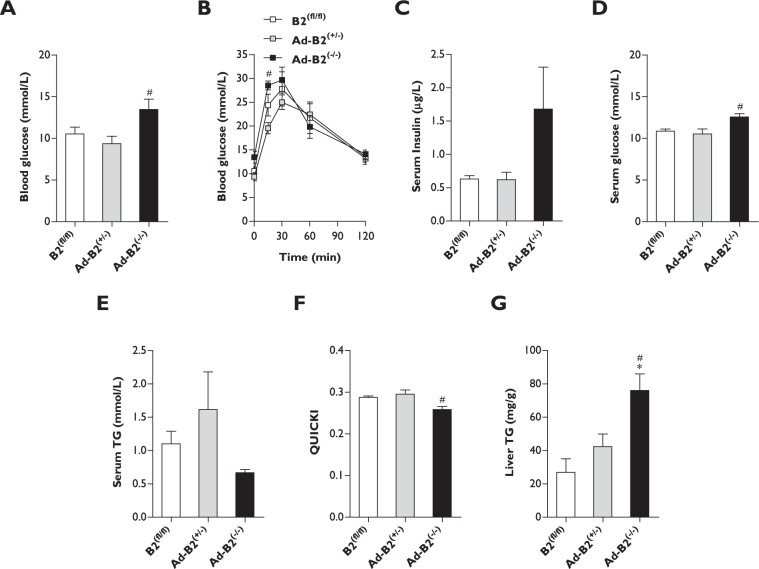
Female Ad-B2^(−/−)^ mice only develop moderate metabolic dysfunction when fed a high-fat diet at thermoneutrality. Five hour fasted blood glucose levels (**A**) and glucose tolerance test (**B**) at twenty-eight weeks of age in B2^(fl/fl)^, Ad-B2^(+/−)^ and Ad-B2^(−/−)^ mice housed at thermoneutrality and fed a high-fat diet for four weeks (n = 5–6). Five hour fasted circulating serum levels of insulin (**C**), glucose (**D**), triglyceride (**E**) and quantitative sensitivity check index analysis (**F**) in twenty-eight week old B2^(fl/fl)^, Ad-B2^(+/−)^ and Ad-B2^(−/−)^ female mice housed at thermoneutrality and fed a high-fat diet for four weeks (n = 5–6). (**G**) Liver triglyceride levels normalised to tissue weight in five hour fasted B2^(fl/fl)^, Ad-B2^(+/−)^ and Ad-B2^(−/−)^ mice housed at thermoneutrality and fed a high-fat diet for four weeks (n = 5–6). All data are presented as the mean ± SEM, *p < 0.05 vs B2^(fl/fl)^, ^#^p < 0.05 vs Ad-B2^(+/−)^.

We next determined if feeding Ad-B2^(−/−)^ female mice a HFD when housed under thermoneutral conditions had had any impact on metabolic health. After four weeks of HFD feeding under thermoneutral conditions, five hour fasted basal blood glucose levels were found to be significantly elevated, but only when compared to Ad-B2^(+/−)^ control mice (Fig. 5A). When glucose tolerance tests were performed, Ad-B2^(−/−)^ female mice had significantly elevated blood glucose levels at 15 minutes after being injected with a glucose bolus, however this was only apparent compared to Ad-B2^(+/−)^ mice (Fig. 5B). No significant differences however were found between any of the genotypes at 30, 60 or 120 minutes post glucose injection. We next examined serum parameters in twenty-eight week old mice that had been fasted for five hours. Circulating insulin levels showed a trend to be elevated in Ad-B2^(−/−)^ female mice, however this failed to reach statistical significance against control female mice (Fig. 5C). Serum glucose levels were again found to be significantly increased in Ad-B2^(−/−)^ mice, but only when compared to Ad-B2^(+/−)^ controls (Fig. 5D). Serum triglyceride levels continued not to be significantly different between any of the genotypes under these conditions (Fig. 5E). QUICKI analysis indicated that Ad-B2^(−/−)^ female mice had significantly lower values compared to Ad-B2^(+/−)^ female mice but not B2^(fl/fl)^ female mice (Fig. 5F). This indicates that female Ad-B2^(−/−)^ mice appear to have developed modest insulin resistance, at least when compared to Ad-B2^(+/−)^ controls. Interestingly, when lipid levels were examined in the livers of these animals, Ad-B2^(−/−)^ female mice had significantly elevated triglyceride levels compared to both B2^(fl/fl)^ and Ad-B2^(+/−)^ control mice (Fig. 5G). This provides further evidence to suggest that Ad-B2^(−/−)^ female mice are unable to safely store dietary lipids in adipocytes as a consequence of lipodystrophy caused by the ablation of *Bscl2* specifically in adipose tissue. Overall, these findings show that adipose specific ablation of seipin in Ad-B2^(−/−)^ female mice causes lipodystrophy, but does not cause the overt metabolic disease observed with congenital loss of seipin in mice or humans. This is similar to our previous findings in male mice. However, here Ad-B2^(−/−)^ female mice were also simultaneously subjected to HFD feeding and thermoneutral housing. Despite a striking inability to expand their adipose mass under these metabolically challenging conditions, they only developed mild dysregulation of glucose metabolism.

## Discussion

Pathogenic mutations that affect the *BSCL2* gene cause the most severe form of congenital generalised lipodystrophy known in humans^6^. Patients with this condition suffer from an almost complete lack of adipose tissue and develop metabolic complications such as hepatic steatosis, hypertriglyceridemia and insulin resistance^29^. We recently reported that adipose tissue-specific ablation of *Bscl2* is sufficient to cause early-onset generalised lipodystrophy in male mice^21^. Surprisingly however, this mouse model failed to develop the severe metabolic dysfunction observed in global *Bscl2* knockout mice^18–21^. Interestingly, clinical reports suggest that sexual dimorphism may exist in conditions of lipodystrophy, as female patients appear to be more severely affected by metabolic complications than male patients, although this has not been examined systematically or experimentally^26,27^. For this reason, we examined whether adipose tissue-specific loss of *Bscl2* in female mice would lead to the development of metabolic dysfunction which had not been apparent in male mice.

Here we show that female Ad-B2^(−/−)^ mice fed a chow diet and maintained at standard housing temperatures (21 °C) develop lipodystrophy as young as six weeks of age, with significantly decreased levels of adiposity and increased lean mass levels. Consistent with alterations in adipose tissue development, significant decreases in mRNA transcripts and circulating serum levels of the adipokines adiponectin and leptin were also apparent. Despite this, female Ad-B2^(−/−)^ mice failed to develop severe metabolic dysfunction under these conditions, similar to the situation we have previously described in male Ad-B2^(−/−)^ mice^21^. Although there were no major differences between genders in terms of metabolic dysfunction, BAT mass was more significantly reduced in females than we had previously found in male mice^21^. In addition, female mice did not display increased expression of markers of thermogenesis in residual GWAT depots, which were evident in EWAT of both global and adipose tissue-specific knockout male mice^19,21,25^. BAT is capable of significant energy dissipation via heat generation due to adaptive thermogenesis^30–32^. Moreover, activation of BAT thermogenesis can improve glucose tolerance both in mice and humans^33,34^. Hence, the greater severity of BAT loss in female Ad-B2^(−/−)^ mice could explain why we find that female mice display modestly dysregulated glycaemic control, which was not apparent in their male counterparts^21^. However, these changes were subtle and glucose tolerance tests in female mice were not significantly altered whilst other parameters of metabolic dysfunction were not changed.

Although significant alterations to BAT mass are apparent in both global and adipose tissue-specific *Bscl2* knockout mice^18–21^, previous studies indicate that loss of *Bscl2* does not impair the brown adipogenic differentiation program per se^35,36^. Indeed, *Bscl2* knockout mice are capable of maintaining their core body temperature when acclimatised to cold temperatures (4 °C) and fed *ad libitum*, indicating that *Bscl2* deficiency is likely to play a non-cell autonomous role in the decreased BAT mass observed in these models^36^. As our female Ad-B2^(−/−)^ mice were initially maintained under standard housing temperatures (21 °C), they may be considered to be under chronic thermal stress and effectively increase their metabolic rate in order to defend their body temperature. We therefore examined female Ad-B2^(−/−)^ mice under thermoneutral conditions (30 °C), which can uncover metabolic phenotypes by reducing lipid and carbohydrate oxidation linked to thermogenesis^28^. Female Ad-B2^(−/−)^ mice did not show any differences in body weight, were lipodystrophic and remained glucose tolerant despite being housed at thermoneutrality for nine weeks. Thus, lipodystrophy as a consequence of adipose-specific *Bscl2* ablation remained insufficient to cause the severe metabolic dysfunction that is observed in global *Bscl2* knockout mouse models^18–21^. To challenge this cohort further, we fed mice a HFD for a period of four weeks. Just as observed in male Ad-B2^(−/−)^ mice^21^, female Ad-B2^(−/−)^ mice resisted high-fat diet induced weight gain due to a failure of adipose tissue expansion. Whilst severe metabolic dysfunction was still not apparent under these conditions, female Ad-B2^(−/−)^ mice did show modest changes, indicative of metabolic complications. Therefore, it is possible to induce symptoms of metabolic dysfunction in Ad-B2^(−/−)^ female mice compared to controls, however, these only become apparent when mice are simultaneously aged under thermoneutral conditions and challenged with a HFD.

*BSCL2* is widely expressed throughout the body^6^, yet it is a commonly held view that all the metabolic consequences of CGL2 can be explained solely by the lack of adipose tissue development. There is evidence to support this, as re-establishing *Bscl2* selectively in adipose tissue of *Bscl2* null mice is capable of restoring significant adipose mass as well as preventing insulin resistance and severe hepatic steatosis^37^. However, this does not exclude the possibility that when adipose tissue is lacking in CGL2, the additional loss of seipin in non-adipose tissues impairs their ability to compensate for this, increasing the severity of metabolic dysfunction. Experiments examining the specific ablation of *Bscl2* in the liver have also revealed that this alone does not lead to the development of a metabolic phenotype, even when mice were fed a HFD^24^. This also appears to support the notion that the loss of adipose tissue is exclusively responsible for metabolic disease in CGL2. However, liver-specific *Bscl2* knockout mice have normal adipose tissue development and functional adipose mass. It is therefore credible that this could protect them from developing metabolic dysfunction.

It is interesting to note that the onset of metabolic complications in our model coincides with significantly elevated liver triglyceride levels in female Ad-B2^(−/−)^ mice compared to control animals. Severe hepatic steatosis is a striking feature found in all *Bscl2* global knockout mice^18–21^ and in patients that suffer from CGL2^4^. Due to the use of the *Adipoq*-Cre driver to ablate *Bscl2* specifically in adipose tissue, *Bscl2* mRNA levels in the liver are unaffected in female Ad-B2^(−/−)^ mice. Consequently, the presence of functional seipin within the liver of our model may offer some protection from the development of severe metabolic dysfunction. For example, in chow fed female Ad-B2^(−/−)^ mice maintained under standard conditions, seipin’s acknowledged role in regulating lipid droplet dynamics may enable dietary lipids to be safely packaged or utilised within hepatocytes, although they would otherwise normally be stored in adipose tissue. However, when challenged sufficiently, e.g. with a HFD at 30 °C, a threshold may be reached where this safe hepatic lipid storage is overwhelmed and metabolic dysfunction begins to develop. In contrast, global *Bscl2* knockout mouse would lack this protective role of seipin within the liver, which could lead to more rapid and harmful lipid accumulation within hepatocytes, regardless of the housing or dietary conditions.

In support of this view, a recent report has highlighted that *Bscl2* does appear to play a cell autonomous role within the liver. Knockdown of *Bscl2* resulted in an increase in both the number and size of intracellular lipid droplets within hepatocytes^38^. Additional evidence is beginning to emerge that *Bscl2* may have other cell autonomous roles in other non-adipose tissues, which could play important roles in metabolic homeostasis. Another recent study has found that global *Bscl2* knockout mice exhibit impaired kidney function, with increased renal lipid deposition and elevations in glycosuria^39^. Adipose tissue transplant partially rescued renal function in *Bscl2* knockout mice, indicating that loss of adipose tissue was at least partly responsible for kidney disease in these mice. However, further studies will be needed in order to confirm whether the loss of *Bscl2* specifically in the kidney also contributes. Previous studies have shown that the hypertrophic cardiomyopathy phenotype observed in *Bscl2* null mice appears to be caused by hyperglycaemia, rather than the loss of *Bscl2* in cardiac tissue^40^. Seipin is also abundantly expressed in some brain regions and neuronal populations associated with the regulation of energy balance including, the paraventricular nucleus of the hypothalamus and dorsal vagal complex in the brainstem^6,41,42^. However, the ability of seipin in specific neuronal populations to influence metabolic health has yet to be probed in detail. The use of alternative tissue-specific knockout mouse models will be needed to dissect the potential roles of seipin in the kidney, brain and other tissues in order to understand its potential importance in metabolic actions outside adipose tissue.

Overall, our findings along with recently published data indicate that *Bscl2* may play important roles in non-adipose tissues that might contribute to the development of severe metabolic dysfunction in CGL2. If this is the case, these previously overlooked tissues could represent novel targets for therapeutic intervention. Indeed this approach is already exemplified by leptin replacement therapy in CGL, where insulin resistance and hepatic steatosis is ameliorated without altering adipose tissue mass^43^. Further efforts should therefore be made to fully understand and characterise the mechanistic role played by seipin in non-adipose tissues. This information could provide new insights regarding how metabolic dysfunction develops in patients with congenital generalised lipodystrophy, but also in individuals suffering from other forms of adipose tissue dysfunction such as in obesity.

## Methods

### Animal studies

*Bscl2* flox’d mice (B2^(fl/fl)^) were generated as previously described^21^. To generate adipocyte-specific seipin knockout mice (Ad-B2^(−/−)^), B2^(fl/fl)^ mice were crossed with heterozygous B2^(fl/+)^ mice also carrying Cre recombinase driven by the *Adipoq* promoter (Adiponectin-Cre). Adiponectin-Cre mice were generously provided by Dr Evan Rosen, Beth Israel Deaconess Medical Centre, Harvard Medical School, Boston, USA. Animal procedures were approved by the University of Aberdeen Ethics Review Board and performed under project licenses (PPL: P94B395EO and PFAD33FA2) approved by the UK Home Office. All experiments used female mice, which were group-housed at standard housing temperatures (21 °C) and exposed to a 12 hr/12 hr light-dark period. For studies performed at thermoneutrality, eleven week old female mice were group-housed at 30 °C and exposed to a 12 hr/12 hr light-dark period. Female B2^(fl/fl)^ mice and mice heterozygous for both Adiponectin-Cre and B2^(fl/f)^ (Ad-B2^(+/−)^) that were littermates to Ad-B2^(−/−)^ mice were used as controls. Mice were always given *ad libitum* access to water and a standard rodent chow diet (CRM (P) 801722, Special Diets Services) unless otherwise stated. Tissues were rapidly dissected post-mortem, frozen in liquid nitrogen then stored at −70 °C.

### Metabolic studies

Fat and lean mass normalised to body weight were measured in mice aged six and twelve weeks (standard housing temperatures) using the EchoMRI™-500 body composition analyser (Zinsser Analytic GmbH) or nineteen weeks of age (thermoneutrality) by dual-energy X-ray absorptiometry (DEXA, Lunar PIXImus). Prior to glucose tolerance tests (GTT), mice were placed into clean cages and food was withheld for five hours. Basal glucose readings (0 min) were determined by glucometer readings (AlphaTrak^®^ II, Zoetisus) from tail punctures. Mice were then given a 2 mg/g d-glucose (Sigma) bolus by intraperitoneal injection. Blood glucose levels were monitored at 15, 30, 60 and 120 minutes. Mice had *ad libitum* access to water throughout. Twenty four-week-old Ad-B2^(−/−)^, Ad-B2^(+/−)^ and *Bscl2*^(fl/fl)^ littermates housed at thermoneutrality were placed on a high-fat diet (60% kcals from fat (D12492), Research Diets) for four weeks. All mice had *ad libitum* access to food and water unless otherwise stated.

### Gene expression

Total RNA was extracted from frozen tissues using the RNeasy mini kit (Qiagen) following the manufactures protocol. Equal quantities of RNA were DNase I treated (Sigma) then reverse transcribed with M-MLV reverse transcriptase, 5X reaction buffer, dNTP’s and random primers (Promega). Real-time quantitative PCR was performed on the 7900HT system (Applied Biosystems) or CFX384 Touch™ Real-Time PCR Detection System (BioRad). NTC and NoRT controls were performed for every gene analysed. The geometric mean of three stable reference genes (*Nono*, *Ywhaz* and *Hprt*) was used for normalisation.

### Serum analysis

Blood was collected from 16 week old chow fed mice (standard housing temperatures) or twenty eight week old HFD fed mice (thermoneutrality) fasted for five hours by cardiac puncture, placed and inverted in SST™ amber tubes (BD Microtainer^®^) and incubated at room temperature for 30 minutes. Samples were then centrifuged at 12,000 × g for 10 minutes and the separated serum collected. Insulin, Adiponectin and Leptin analysis was performed at the Core Biochemical Assay Laboratory (Cambridge, UK). Glucose levels were determined using the Glucose Colorimetric Assay Kit (Cayman Chemical) and following the manufacturer’s protocol provided. Serum triglyceride levels were determined using the Triglyceride Liquid Assay (Sentinel Diagnostics) following the manufacturer’s instructions. Quantitative insulin sensitivity check index (QUICKI) was calculated from fasting glucose (mg/dL) and insulin (µU/mL) values as previously described^44^. QUICKI = 1/[log(I_0_) + log(G_0_)], where I_0_ is fasting insulin and G_0_ is fasting glucose. QUICKI is a dimensionless index without units.

### Liver TG assay

Frozen liver tissue samples were weighed and then homogenised in 1 ml of PBS. Samples were kept on ice at all times. Liver lysates were centrifuged at 12,000 × g for 10 minutes at 4 °C. The supernatant was collected and triglyceride (TG) levels were determined using the Triglyceride Liquid Assay (Sentinel Diagnostics) following the manufacturer’s instructions. TG levels were then normalised to individual tissue weights.

### Statistical analyses

All data are presented as the mean ± SEM and were analysed by one-way analysis of variance with Tukey post-hoc test or two-way repeated measures analysis of variance with Bonferroni post-hoc test as appropriate using GraphPad Prism. A *P*-value < 0.05 was considered as statistically significant.

## Electronic supplementary material

Supplementary Material

## Data Availability

The datasets generated during and/or analysed during the current study are available from the corresponding author on reasonable request.
