# Supplementary material for: Female adipose tissue-specific Bscl2 knockout mice develop only moderate metabolic dysfunction when housed at thermoneutrality and fed a high-fat diet
Source: Sci Rep. 2018 Dec 14;8:17863. doi: 10.1038/s41598-018-36078-9 (PMC6294754; doi:10.1038/s41598-018-36078-9)
Supplement: Supplementary file 1 — Supplementary Material [file 41598_2018_36078_MOESM1_ESM.pdf]

Female adipose tissue-specific *Bscl2* knockout mice develop only moderate metabolic dysfunction when housed at thermoneutrality and fed a high-fat diet

George D. Mcilroy, Sharon E. Mitchell, Weiping Han, Mirela Delibegović, Justin J. Rochford

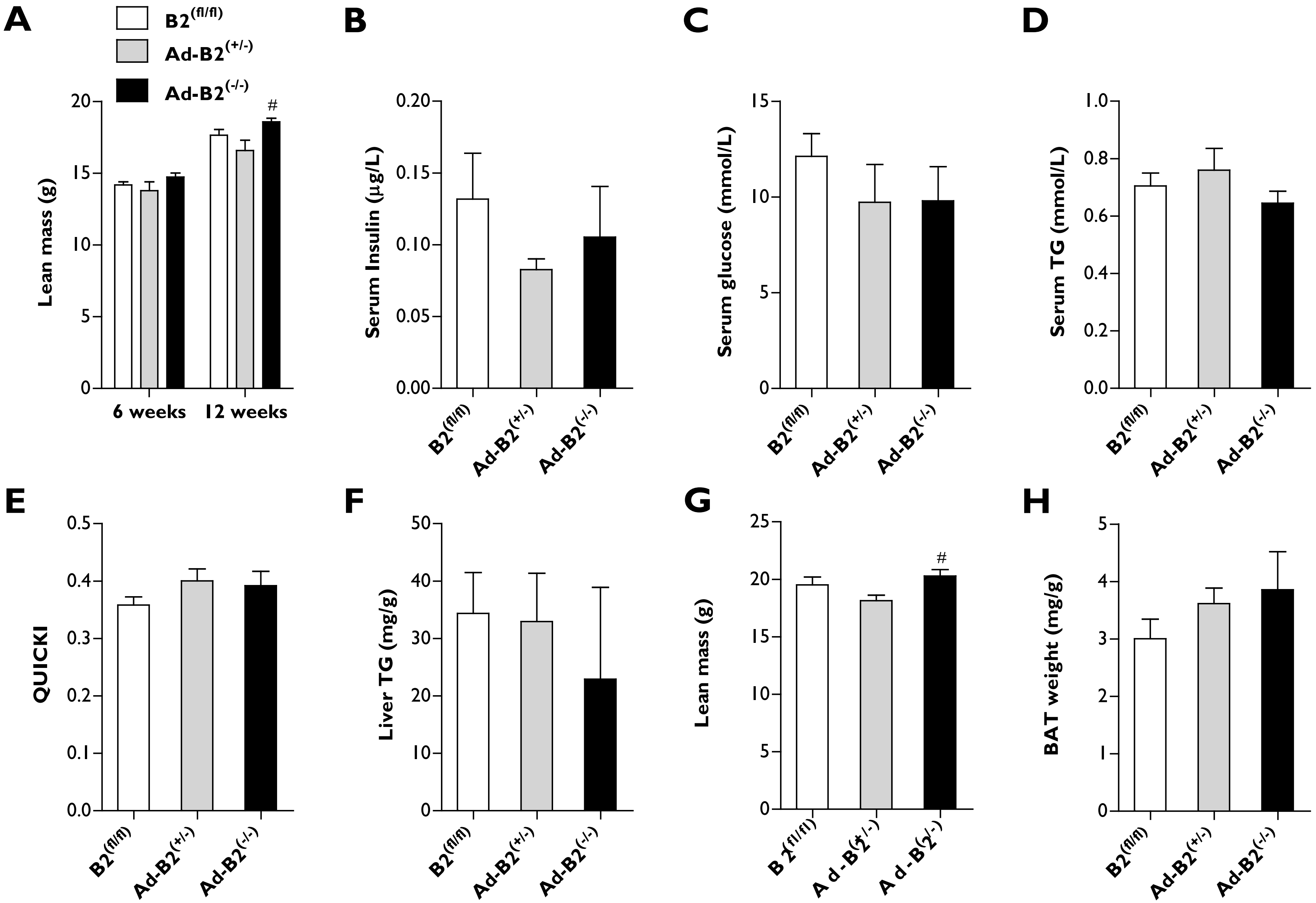

Female adipose tissue-specific *Bscl2* knockout mice develop only moderate metabolic dysfunction when housed at thermoneutrality and fed a high-fat diet

George D. Mcilroy, Sharon E. Mitchell, Weiping Han, Mirela Delibegović, Justin J. Rochford

Supplementary figure 1. (**A**) Absolute lean mass levels assessed by Echo-MRI in B2$^{(fl/fl)}$, Ad-B2$^{(+/-)}$ and Ad-B2$^{(-/-)}$ mice at six and twelve weeks of age (n = 4-8), #p<0.05 vs Ad-B2$^{(+/-)}$. Five hour fasted circulating serum levels of insulin (**B**), glucose (**C**), triglyceride (**D**) and quantitative sensitivity check index analysis (**E**) in sixteen week old B2$^{(fl/fl)}$, Ad-B2$^{(+/-)}$ and Ad-B2$^{(-/-)}$ female mice housed at standard housing temperatures (21°C) and fed a chow diet (n = 4). (**F**) Liver triglyceride levels normalised to tissue weight in five hour fasted B2$^{(fl/fl)}$, Ad-B2$^{(+/-)}$ and Ad-B2$^{(-/-)}$ at sixteen weeks of age (n = 4). (**G**) Absolute lean mass levels assessed by DEXA in B2$^{(fl/fl)}$, Ad-B2$^{(+/-)}$ and Ad-B2$^{(-/-)}$ mice after eight weeks at thermoneutrality (n = 5-6), #p<0.05 vs Ad-B2$^{(+/-)}$. (**H**) BAT weight normalised to body weight in twenty-eight week old B2$^{(fl/fl)}$, Ad-B2$^{(+/-)}$ and Ad-B2$^{(-/-)}$ female mice housed at thermoneutrality (30°C) and fed a high-fat diet for four weeks (n = 4-6). All data are presented as the mean ± SEM.
